# Deformation of the median nerve at different finger postures and wrist angles

**DOI:** 10.7717/peerj.5406

**Published:** 2018-08-09

**Authors:** Ping Yeap Loh, Wen Liang Yeoh, Hiroki Nakashima, Satoshi Muraki

**Affiliations:** 1 Department of Human Science, Faculty of Design, Kyushu University, Fukuoka, Japan; 2 Department of Human Science, Graduate School of Design, Kyushu University, Fukuoka, Japan

**Keywords:** Carpal tunnel, Tendon gliding exercise, Median nerve cross-sectional area, Median nerve diameters

## Abstract

**Background:**

The objective of this study was to evaluate the changes of the median nerve cross-sectional area (MNCSA) and diameters of the median nerve at different finger postures and wrist angles.

**Methods:**

Twenty-five healthy male participants were recruited in this study. The median nerve at wrist crease was examined at six finger postures, and repeated with the wrist in 30° flexion, neutral (0°), and 30° extension. The six finger postures are relaxed, straight finger, hook, full fist, tabletop, and straight fist.

**Results:**

The main effects of both finger postures and wrist angles are significant (*p* < 0.05) on changes of the MNCSA. Different finger tendon gliding postures cause a change in the MNCSA. Furthermore, wrist flexion and extension cause higher deformation of the MNCSA at different finger postures.

**Discussion:**

The median nerve parameters such as MNCSA and diameter were altered by a change in wrist angle and finger posture. The results may help to understand the direct biomechanical stresses on the median nerve by different wrist-finger activities.

## Introduction

Carpal tunnel syndrome (CTS) is a compression neuropathy that affects the median nerve at the wrist region. The median nerve lies beneath the transverse carpal ligament and is vulnerable to biomechanical stress. Various studies suggest that workplace biomechanical factors such as hand/wrist posture and hand force are associated with the incidence of CTS (Harris-Adamson et al., 2015; Bao et al., 2016). Several studies using ultrasound imaging techniques to investigate the mobility (Martínez-Payá et al., 2015) and morphological characteristics of the median nerve among healthy and CTS patients have been conducted (Werner, Jacobson & Jamadar, 2004; Hunderfund et al., 2011; Korstanje et al., 2012; Yoshii et al., 2013). It was observed that active differential finger motions among CTS patients cause a decrease of the median nerve cross-sectional area (MNCSA) and perimeter in contrast to the control group (Van Doesburg et al., 2012; Yoshii, Ishii & Sakai, 2013).

The tendons of the extrinsic finger flexor muscles such as the flexor digitorum superficialis (FDS), flexor digitorum profundus (FDP), and flexor pollicis longus pass through the carpal tunnel and control the flexion of interphalangeal (IP) and metacarpophalangeal (MCP) joints. Active finger flexion results in the gliding of both finger flexor tendons and median nerve at different amplitudes in association with specific finger positions, such as the tabletop, hook, and full fist position (Wehbé, 1987; Lopes et al., 2011). In addition, the median nerve and flexor tendons are connected by subsynovial connective tissues. These are loose connective tissues that enable differential gliding between the tendons and median nerve during wrist and finger motions. However, the shear strain of the synovial connective tissue between the median nerve and tendons increases with repetitive finger motions (Tat, Kociolek & Keir, 2013). Forceful and prolonged finger movements may lead to inflammation of the subsynovial connective tissues, resulting in higher compression stress on the median nerve and lesser nerve excursion during finger movements (Keir & Rempel, 2005).

**Figure 1:**
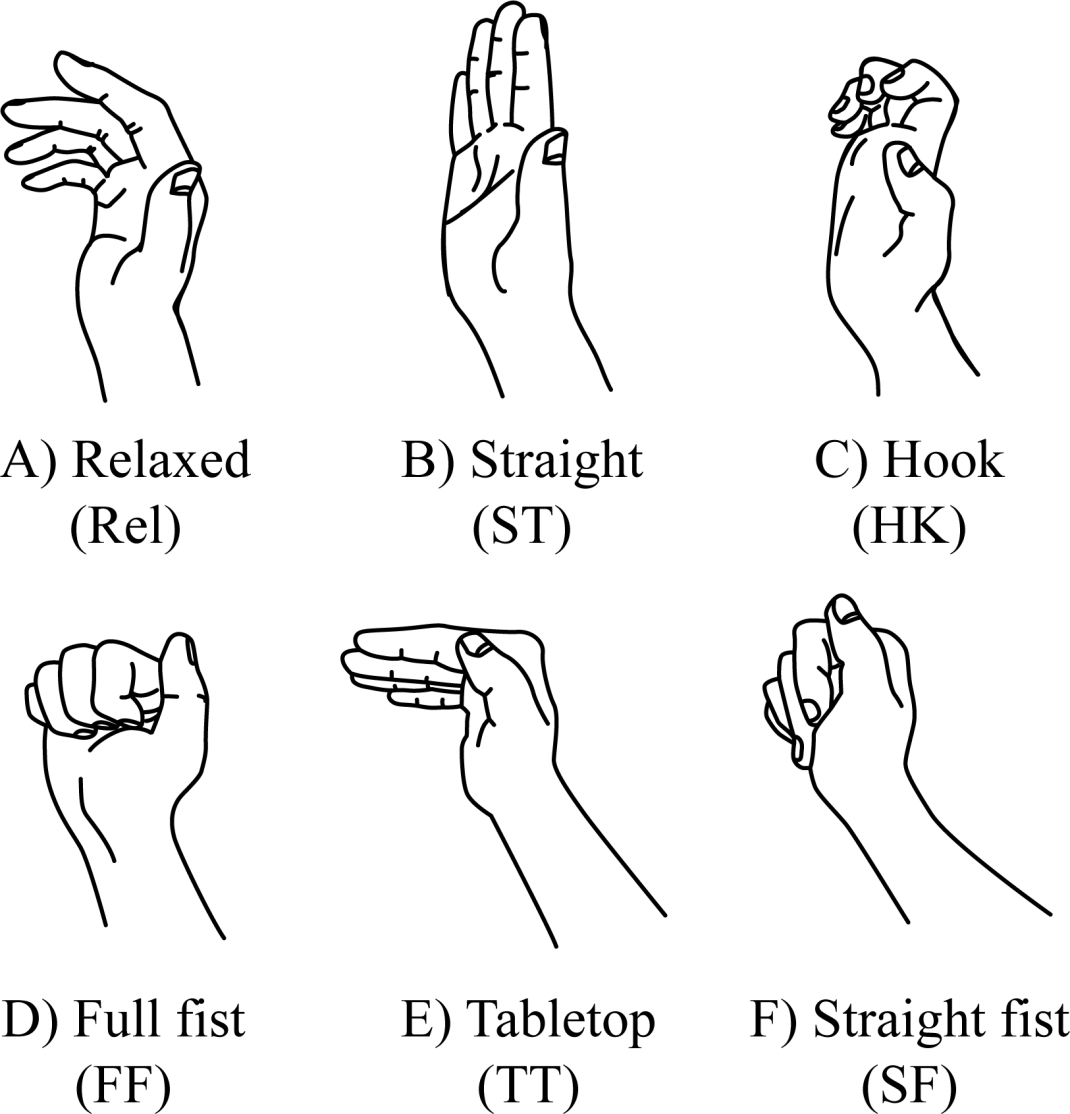
Relaxed fingers and finger tendon gliding positions. (A) Relaxed, (B) straight, (C) hook, (D) full fist, (E) tabletop, (F) straight fist.

The contributions of the extrinsic flexors and intrinsic muscles in finger joint flexion differ in each finger posture (Fig. 1). Intrinsic muscles of the hand are muscles originating from the metacarpal bones or FDP tendons within the hand and inserting to the dorsal extensor expansion of the fingers. The tendons glide distally when moving from a relaxed to straight finger position. Subsequently, the tendons glide proximally as the MCP and IP joints move into flexion position. Different finger postures result in various tendon gliding amplitudes. The FDS and FDP achieve maximal gliding in the straight fist (28 mm) and full fist (34 mm) position, respectively; hook position results in maximal gliding between FDS and FDP (11 mm) (Wehbé & Hunter, 1985). Similarly, the intrinsic muscles contribute to MCP joint flexion to achieve tabletop, full fist, and straight fist along with the flexion of the IP joints. The displacement of finger flexor tendons during finger motions happens in proximo-distal, radial-ulnar, and dorsal-palmar directions. Individual or multi-finger motions cause geometric displacement of the finger flexor tendons which leads to deformation of the median nerve (Ugbolue et al., 2005; Canuto et al., 2006; Yoshii et al., 2008). In addition, intrusion of lumbrical muscles into carpal tunnel during forceful finger flexion may lead to median nerve deformation (Cartwright et al., 2014).

Recent studies suggest that the median nerve can be deformed when the wrist position changes from neutral to flexion or extension among the young and the elderly (Wang et al., 2014; Loh & Muraki, 2015; Loh, Nakashima & Muraki, 2015). Generally, a moving joint causes nerve bed elongation and nerve excursion; this elongation stress and transverse contraction of the nerve result in a decrease of the nerve cross-sectional area (Szabo et al., 1994; Millesi, Zoch & Reihsner, 1995; Topp & Boyd, 2006). Therefore, the combination of wrist and finger movements may lead to a higher deformation of the median nerve. The primary objective of this study was to investigate the morphological changes of the median nerve, namely, the MNCSA and diameter of the median nerve in the radial-ulnar direction (D1) and dorsal-palmar direction (D2) of each finger posture at different wrist angles. Our main hypotheses were that the MNCSA and median nerve diameter are associated with different finger postures and the wrist angle deviation from the neutral position leads to higher deformation of the median nerve measurements.

## Methods

Twenty-five healthy male participants (Table 1) without known upper limb musculoskeletal pathology were recruited for this study. Informed and written consent was obtained from the participants. Edinburgh Handedness Inventory was used to determine the handedness of the participants (Oldfield, 1971). This study was approved by the Ethics Committee of the Faculty of Design, Kyushu University (Approval Number 141).

**Table 1:** Characteristics of the participants (*n* = 25).

	Mean ± SD
Age (years)	24.0 ± 1.8
Height (cm)	171.1 ± 4.8
Weight (kg)	64.0 ± 8.1
BMI (kg/m^2^)	21.7 ± 1.7
Handedness (Right : left hand dominant)	24 : 1

### Ultrasound examination protocol

The LOGIQ e ultrasound system (GE Healthcare, Chicago, IL, USA) equipped with a 12L-RS transducer (imaging frequency bandwidth of 5–13 MHz) was used in this study. The ultrasound transducer acquisition was optimized for a frequency of 12 MHz and a depth of 4.5 cm. A 7.0-mm-thick sonar pad (Nippon BXI Inc., Tokyo, Japan) was used as a coupling agent during the ultrasound examination to minimize wrist compression and to ensure a good acoustic contact between the ultrasound transducer and the skin. The examiner placed the ultrasound transducer gently on the sonar pad to minimize the pressure on the wrist throughout the examination. The ultrasound transducer was placed perpendicularly to the wrist and parallel to the proximal wrist crease to identify the median nerve in the transverse plane at the proximal carpal tunnel. The median nerve was identified at the superficial level by the hyperechogenic rim containing hypoechogenic nerve fascicles (Kele, 2012).

The participants were seated with the forearm resting on an arm support on the table. The elbow rested at 30° flexion with the forearm supinated during the ultrasonography examination. Before the beginning of the assessment, the examiner used a 180° wrist goniometer to determine the wrist angle. First, the axis point of the goniometer was positioned at the triquetrum. Then, the static arm and moveable arm of the goniometer were placed parallel to the ulnar bone and the fifth metacarpal bone to determine the wrist angle. Six finger postures were examined as follows:

(1) relaxed, the fingers rest in natural curvature and without active flexion and/or extension (Fig. 1A)
(2) straight finger, 0° extension of MCP joint (MCPJ), proximal interphalangeal joint (PIPJ), and distal interphalangeal joint (DIPJ) (Fig. 1B)
(3) hook, 0° extension of MCPJ with full flexion of PIPJ and DIPJ (Fig. 1C)
(4) full fist, full flexion of MCPJ, PIPJ, and DIPJ (Fig. 1D)
(5) tabletop, 90° flexion of MCPJ and 0° extension of PIPJ and DIPJ (Fig. 1E)
(6) straight fist, full flexion of MCPJ and PIPJ and 0° extension of DIPJ (Fig. 1F)

The participants practiced maintaining the finger postures before the ultrasonography examination to ensure the MCP and IP joint angle at each finger posture. The wrist angle was determined using a goniometer prior to the ultrasonographic examination; the participants were asked to hold the wrist at designated angles (neutral 0°, 30° flexion, or 30° extension) and maintain the finger posture (Fig. 1) with minimal effort while the examiner placed the ultrasound transducer on the proximal wrist crease. Images were taken as the participant held the designated wrist angle and finger posture for at least 3 s. The wrist angles were maintained by the participants during the examination process without the examiner’s assistance. The ultrasound examination for each finger posture at different wrist angles was repeated three times for both the dominant and non-dominant hands; the finger and wrist postures were repositioned between each examination.

### Images processing and analysis

**Figure 2:**
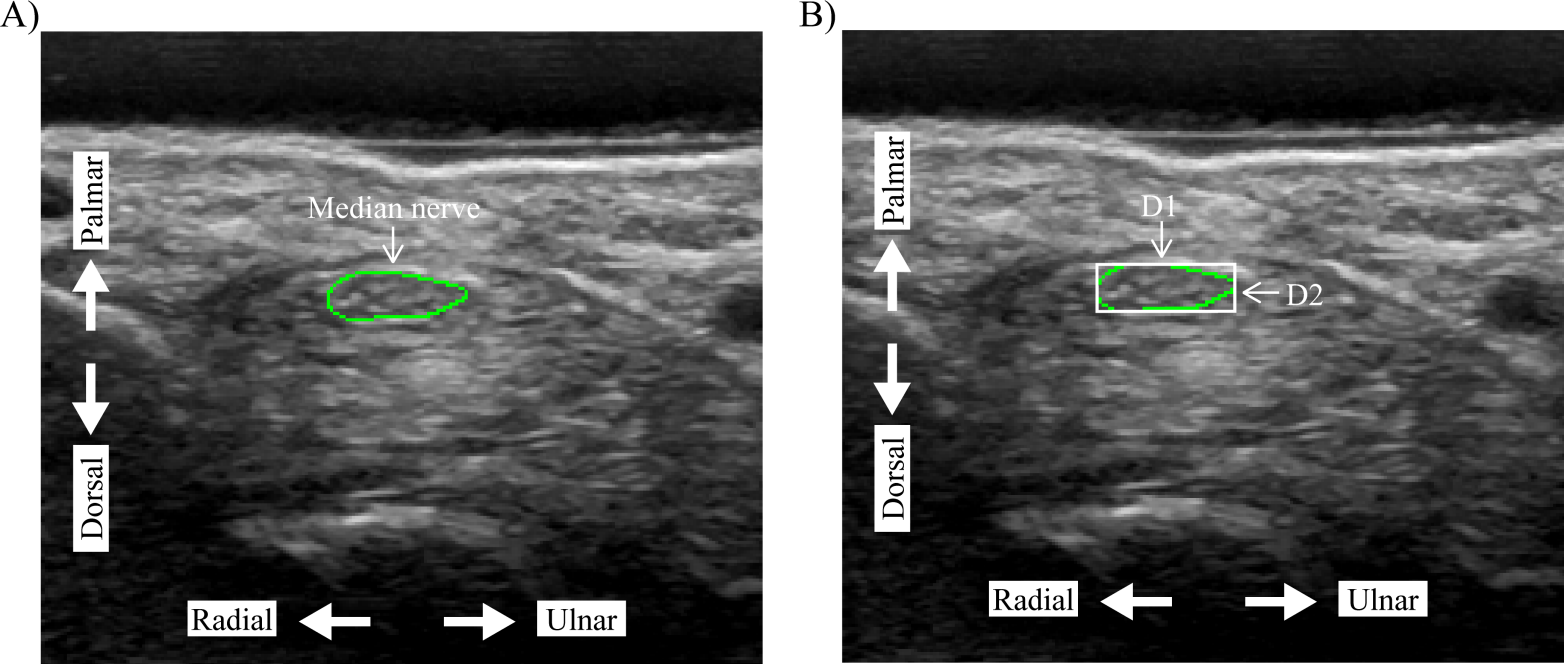
Quantification of the (A) median nerve cross-sectional area (MNCSA) by tracing method, and (B) diameters of median nerve, D1 and D2, by minimum bounding rectangle method. D1, diameter in radial-ulnar direction; D2, diameter in dorsal-palmar direction.

The recorded images by LOGIQ e ultrasound system were then exported and randomized prior to the image analysis process. The examiner was blinded about the wrist angle and finger posture during the image analysis process. First, the examiner traced the median nerve along the hyperechogenic rim using ImageJ (Fig. 2A) (Duncan, Sullivan & Lomas, 1999; Schneider, Rasband & Eliceiri, 2012). Our previous study using this quantifying method showed good to excellent inter- and intra-rater reliability (Loh & Muraki, 2015). After tracing the outline of the median nerve, the OpenCV library (version 3.1) accessed with a python script was used to quantify the D1 (radial-ulnar direction) and D2 (dorsal-palmar direction) using the minimum bounding rectangle method (Fig. 2B). The average of three images was calculated for the MNCSA, D1, and D2 at each finger posture. The deformation percentages of the MNCSA, D1, and D2 were calculated with the following equation:

\begin{eqnarray*}\text{Deformation Percentage}\nonumber\\\displaystyle \quad =  \frac{\text{Measurement at different finger posture} - \text{Measurement at relaxed finger posture at neutral wrist}}{\text{Measurement at relaxed finger posture at neutral wrist}}  \times 100\text{%} \end{eqnarray*}

### Statistical analysis

Statistical analysis was performed using SPSS version 21.0 software (IBM Corporation, Chicago, IL, USA). The sample characteristics of MNCSA in the dominant and nondominant hands were examined with the Shapiro–Wilk normality test.

A two-way repeated analysis of variance (6 × 3 factorial design) was conducted with six finger postures, three wrist angles (30° flexion, neutral (0°), and 30° extension), as factors to examine differences in MNCSA, D1, and D2 for both dominant and nondominant hand. The post-hoc pairwise Bonferroni-corrected comparison was used to examine the significant effects. Significance was set at *α* = 0.05. All results, including tables and figures, are presented in mean ± standard deviation.

## Results

### Effect of the finger posture and wrist angle on the change in MNCSA

**Figure 3:**
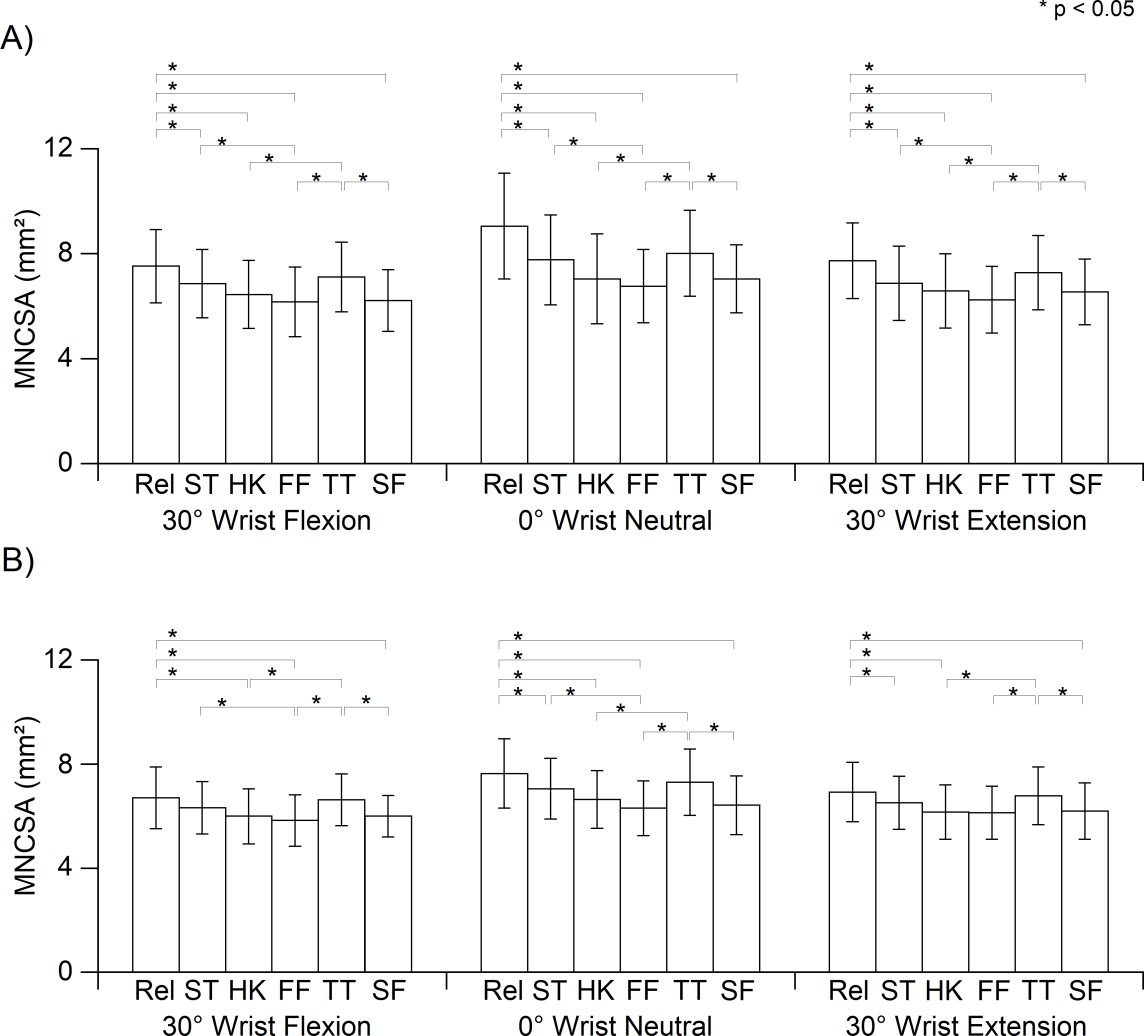
Median nerve cross-sectional area (MNCSA) of each finger position at (A) dominant hand and (B) nondominant hand. Relaxed (Rel), straight finger (ST), hook (HK), full fist (FF), tabletop (TT), straight fist (SF).

The main effects of the finger postures (*p* < 0.05) and the wrist angles (*p* < 0.05) on the changes of MNCSA were significant for both hands. Significant interaction was found for finger posture × wrist angle (both hands, *p* < 0.05). The MNCSA at the relaxed finger was larger in contrast to that in the other finger postures (Fig. 3) for both the dominant and non-dominant hands. The MNCSA at full fist was the smallest among all finger postures. Subsequently, the MNCSA at the same finger posture in 30° flexion and 30° extension of the wrist was smaller in contrast to that for a neutral wrist (0°). Although the wrist angle in 30° flexion and 30° extension caused a greater change in the MNCSA when compared to the neutral wrist (0°), similar deformation percentages were observed for the same finger posture at different wrist angles in compared to relaxed finger at neutral wrist (0°).

### Effect of the finger posture and wrist angle on the changes in D1 and D2

**Figure 4:**
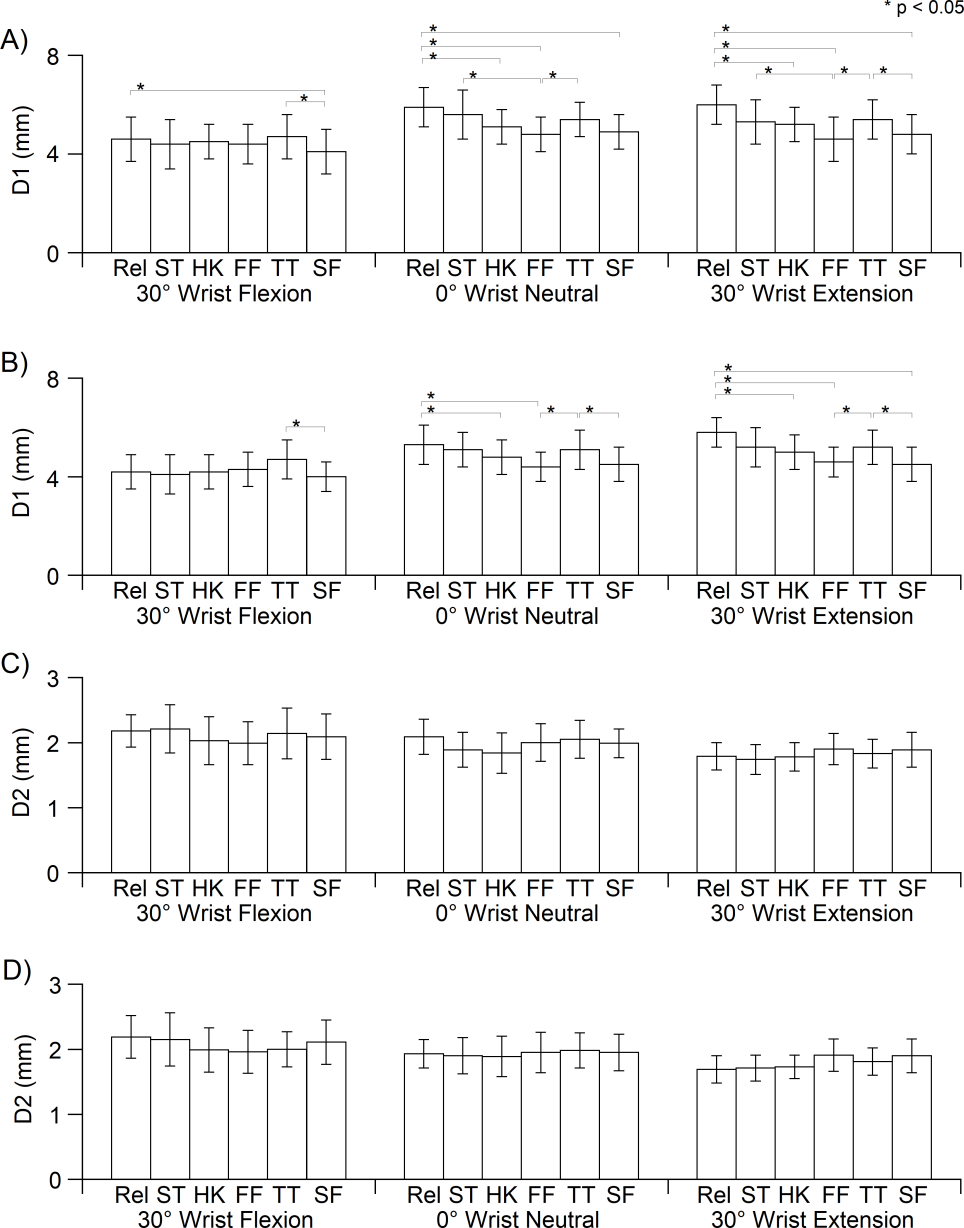
Median nerve diameters D1 and D2 of each finger position at dominant (A, C) and nondominant hand (B, D). D1, diameter in radial-ulnar direction; D2, diameter in dorsal-palmar direction; Rel, Relaxed; ST, straight finger; HK, hook; FF, full fist; TT, tabletop; SF, straight fist.

The wrist angle significantly affected the median nerve diameter in both hands (D1, *p* < 0.05; D2, *p* < 0.05). In addition, there was a significant difference in the main effect of finger posture on the changes of the D1 (both hands, *p* < 0.05). Additionally, the finger posture × wrist angle interaction was not significant for the changes of D1 and D2 for both hands. At neutral wrist, D1 at different finger postures were generally shorter in contrast to that for the relaxed finger (Figs. 4A and 4B).

With reference to D1 of the relaxed finger in a neutral wrist position, the deformation percentages of D1 at each finger posture are approximately −23%, except for that in the tabletop position (−13%) (Table 2). D1 in the tabletop position was longer for all finger postures. In addition, D2 generally became smaller than that in the relaxed finger, and full fist caused the highest deformation of D2 (Figs. 4C and 4D, Table 2). Although the interaction did not reach significance level, D1 became smaller as the position changed from a relaxed finger to a different posture, and D1 at full fist was the shortest in contrast to that for other finger postures (Figs. 4A and 4B). Notably, wrist extension caused a different deformation trend of D2 in contrast to the neutral wrist and wrist at 30° flexion. D2 at the relaxed finger is the shortest among all finger postures. The D2 length became longer as the relaxed finger changed to different finger postures, and the D2 lengths at full fist and straight fist were longer than that in the other finger postures (Figs. 4C and 4D, and Table 2).

**Table 2:** Deformation percentage (%) of the median nerve cross-sectional area and diameters of each finger posture at different wrist angles.

Wrist	Wrist angle	Finger postures					
		Relaxed	Straight	Hook	Full fist	Tabletop	Straight fist
**Median nerve cross-sectional area (MNCSA, mm^2^)**							
Dom	30° Flex	−16.2	−23.6	−28.3	−31.6	−20.7	−30.5
	0° Neutral	–	−14.1	−21.8	−25.0	−11.0	−21.5
	30° Ext	−13.8	−23.7	−26.8	−30.6	−18.9	−27.0
NonDom	30° Flex	−12.2	−16.9	−21.3	−23.3	−12.7	−20.7
	0° Neutral	–	−7.4	−12.8	−17.3	−4.3	−15.7
	30° Ext	−9.1	−14.2	−19.2	−19.5	−10.9	−18.6
**Diameter in radial-ulnar direction (D1, mm)**							
Dom	30° Flex	−21.8	−26.3	−25.8	−27.8	−21.2	−30.8
	0° Neutral	–	−6.5	−12.3	−20.5	−10.1	−17.5
	30° Ext	4.1	−8.7	−14.7	−20.0	−7.7	−21.0
NonDom	30° Flex	−23.5	−23.1	−21.6	−20.2	−13.2	−23.5
	0° Neutral	–	−4.7	−10.2	−16.8	−4.9	−16.3
	30° Ext	9.4	−1.9	−8.3	−16.0	−4.1	−16.8
**Diameter in dorsal-palmar direction (D2, mm)**							
Dom	30° Flex	6.3	4.5	−2.5	−5.1	1.4	1.9
	0° Neutral	–	−8.9	−11.2	−4.5	−1.4	−4.9
	30° Ext	−19.7	−16.9	−15.2	−12.0	−12.4	−7.5
NonDom	30° Flex	14.3	10.6	2.8	−1.8	1.8	6.4
	0° Neutral	–	−2.1	−1.9	0.8	1.5	0.5
	30° Ext	−18.0	−12.3	−10.9	−2.8	−6.4	−0.7

**Notes.**

Dom dominant wrist NonDom nondominant wrist Flex Flexion Ext Extension

## Discussion

The carpal tunnel is a confined space, and the median nerve is unavoidably compressed due to the excursion and displacement of the tendons during finger movements. This study demonstrates the median nerve deformation, specifically MNCSA, D1, and D2, resulting from various finger positioning at different wrist angles. In addition to the absolute changes of the MNCSA, D1, and D2, we calculated the deformation percentage to present the changes of the median nerve associated with finger tendon gliding at different wrist angles.

First, we examined the effect of different finger postures on the MNCSA changes at the neutral (0°) wrist position. Previous studies suggested that finger flexors tendons and median nerve move concurrently and differentially within the carpal tunnel and finger motions cause median nerve deformation (Ugbolue et al., 2005; Van Doesburg et al., 2012). The extension of MCP and IP joints as the relaxed finger changes to a straight finger position can result in transverse contraction and elongation of the median nerve that causes the reduction of the MNCSA. Previous studies suggest that the intrinsic muscles are the main flexors of the MCP joint, and the proximal-distal displacements of FDS and FDP resulting from flexion of the MCP joints are less in contrast to the hook, straight fist, and full fist position (Wehbé & Hunter, 1985; Ugbolue et al., 2005; Koh et al., 2006). Therefore, the radial-ulnar displacements and lower excursion amplitude of the finger flexor tendons in straight finger and tabletop postures may be associated with lower deformation on the MNCSA during active finger flexion movements.

FDS and FDP are the primary muscles that initiate the IP joint flexion in the hook and straight fist positions, whereas maximal excursion of the FDS and FDP is achieved at the full fist position (Wehbé, 1987). Longer excursions of the finger flexor tendons at the hook and straight fist position may cause higher transverse contraction and tensile stress to the median nerve than the straight finger and tabletop position. A larger excursion of the finger flexor tendon while making a full fist could possibly lead to a larger deformation percentage of the MNCSA than that in a hook and straight fist position as observed in this study. A previous study reported the intrusion of the lumbrical muscles (16.6 ± 18.5 mm^2^) into the distal carpal tunnel with full active wrist flexion and full active finger flexion among non-CTS participants (Cartwright et al., 2014). Therefore, an increase in the carpal tunnel pressure from the incursion of the lumbricals may result in a higher deformation ratio of the MNCSA.

Additionally, we examined the effects of the wrist angle changes on the median nerve deformation at each finger tendon gliding posture. The wrist in 30° flexion and 30° extension caused the MNCSA to be further deformed at all finger postures (Table 2). The present study demonstrates that finger flexor tendon gliding during wrist flexion causes the highest deformation of the MNCSA, followed by wrist extension. This may be caused by the tendon and dorsal-palmar displacement of the median nerve in the carpal tunnel at wrist flexion and extension which deviates from that in the neutral position (Canuto et al., 2006; Mogk & Keir, 2008).

We further analyzed the changes of D1 and D2 at the neutral wrist position to understand the deformation of the median nerve in response to each finger posture. The loose space within the epineural tube allows the peripheral nerve to adapt to biomechanical stress such as tensile and compressive stress or a combination of these (Millesi, Zoch & Reihsner, 1995; Keir & Rempel, 2005; Topp & Boyd, 2006; Kociolek, Tat & Keir, 2015). The displacements of finger flexor tendons during wrist and finger movements are a combination of both D1 and D2 and are not uniform (Keir & Wells, 1999). As a result of the tendons’ displacement within the carpal tunnel, the shape of the median nerve changes during wrist and finger movements (Van Doesburg et al., 2012; Yoshii et al., 2013).

On the other hand, changes of finger posture at wrist 30° flexion and extension causes different deformation patterns of D1 and D2 (Fig. 4). Finger movements during wrist extension could lead to a higher compression stress that results in greater deformation percentages of D2 (Table 2). However, the D2 length gradually increases as the finger posture changes from straight to other finger postures at wrist extension (Fig. 4). The observed deformations of D1 and D2 at different finger postures may be closely related to the geometric displacement of the flexor tendons inside the confined carpal tunnel.

The limitations of our study include the angle of the ultrasound transducer and the examination location. The ultrasound transducer was checked and placed perpendicularly to the wrist crease at all wrist angles during the examination. However, the angle of the ultrasound transducer could have changed slightly as the participants actively held the wrist and finger postures. Tilting of the ultrasound transducer towards the wrist crease results in an oblique cross-sectional image of the carpal tunnel, which affects the accuracy of the quantification of the median nerve. Subsequently, we examined the median nerve at the wrist crease in this study. Greater median nerve deformation may occur at the mid-carpal tunnel or distal edge of the carpal tunnel due to different carpal tunnel inlet pressures. In addition, because the holding force while maintaining finger postures was not standardized, the absolute changes of the median nerve may be affected by individual differences. The muscle force of the full finger flexor muscles and wrist at flexion angle is the influential variable that should be taken into consideration, as it could affect the intrusion of lumbricals into the carpal tunnel. Generally, no well-defined lumbrical muscles were observed from our recorded images. Lastly, the deformation trend of the median nerve may vary in different age groups, gender, and depend on the presence of CTS.

## Conclusion

An understanding of the mechanical stress during wrist and finger joint movements on the median nerve is essential for the prevention of CTS. This study demonstrated the effects of various finger positions on the deformation of the median nerve based on a two-dimensional plane. Wrist flexion or extension, by itself or in combination with different finger postures, also caused a reduction of MNCSA. In addition, D1 and D2 decreased during wrist flexion and wrist extension, respectively. Median nerve parameters such as MNCSA and diameter may be useful to understand the direct biomechanical stress on the median nerve based on different wrist and finger activities. Additionally, the obtained parameters may be used as a reference value to conduct comparative studies for different age groups, high-risk populations, and in patients with CTS.

##  Supplemental Information

10.7717/peerj.5406/supp-1 Supplemental Information 1 Supplemental dataset

## Additional Information and Declarations

### Data Availability

The following information was supplied regarding data availability: The raw data are provided in Supplemental Information 1.
